# Metabolic assessment of cerebral palsy with normal clinical MRI using ^18^F-FDG PET imaging: A preliminary report

**DOI:** 10.3389/fneur.2022.844911

**Published:** 2022-09-15

**Authors:** Ruimin Wu, Yan Gao, Huaqiong Zhang, Yijia Chen, Fan Tan, Daobing Zeng, Huabing Wan, Yi Yang, Jiaowei Gu, Zhijun Pei

**Affiliations:** ^1^Department of Nuclear Medicine and Institute of Anesthesiology and Pain, Taihe Hospital, Hubei University of Medicine, Shiyan, China; ^2^Department of Nursing, Hubei University of Medicine, Shiyan, China; ^3^Department of Pediatrics, Hubei University of Medicine, Shiyan, China; ^4^Hubei Key Laboratory of Embryonic Stem Cell Research, Shiyan, China

**Keywords:** cerebral palsy, FDG-PET, brain metabolism, GMFCS scores, diagnosis

## Abstract

To explore the cerebral metabolic patterns of cerebral palsy (CP) patients without structural abnormalities by brain magnetic resonance imaging (MRI) scans, we evaluated ^18^F-fluoro-deoxyglucose positron emission tomography (^18^F-FDG PET) imaging features in patients. Thirty-one children with CP [Gross Motor Function Classification System (GMFCS) levels II-V] showing no structural abnormalities by MRI were enrolled in this study. Regional glucose metabolic activity values were calculated using Scenium software and compared between the right and left cerebral hemispheres. These comparisons revealed asymmetric metabolic reductions in the central region, cerebellum, frontal lobe, and parietal lobe (*p* < 0.01). We next determined whether averaged brain metabolic activity values in different brain regions correlated with GMFCS levels. The metabolic activity values of basal ganglia, left temporal lobe, and cerebellum correlated negatively with GMFCS scores (all *p* < 0.05). This method was applied to the left cerebellum, which showed higher metabolic activity values than those in the right cerebellum in most patients (83.8%), and these values also correlated negatively with GMFCS scores (Spearman's *r* = −0.36, *p* = 0.01). Differential cortical glucose metabolism by ^18^F-FDG PET, may help to distinguish between different CP diagnoses that are not detected by MRI.

## Introduction

Cerebral palsy (CP) is a permanent, non-progressive neurological disorder that is typically characterized by physical disabilities. The majority of patients with CP have motor disabilities including abnormal movement and posture, and cognitive and speech impairments, along with abnormal perceptual symptoms (1). Most cases of CP in children can be attributed to a transient adverse event, such as a preterm birth or non-progressive brain lesions at or around the time of birth. These cases are mainly characterized by CP, which comprises a mixture of dystonic, athetoid, and ataxic features (2). The Gross Motor Function Classification System (GMFCS) is used to classify the severity of motor function deficits in patients with CP, and is graded from 5 (“bad”) to 1 (“good”) (3, 4). Individuals with CP and their families have lower qualities of life than those of the general population (5).

Clinical diagnosis of CP is primarily based on identifying symptoms of motor and cognitive impairments in combination with etiological data and pathological examination. In the last decade, several studies have explored the structural and functional imaging correlates of impairments in social cognition (6–9). Cerebral morphology has been evaluated using radiological imaging methods, especially computed tomography (CT) and magnetic resonance imaging (MRI) (10–12). However, no specific morphological abnormalities have been identified using these structural imaging methods for the earlier stages of CP. Functional MRI has been used to diagnose specific clinical forms of CP in children (13), but it has reported that some children with CP have no abnormalities in their MRI scans (14).

Metabolic activity in the brain can be measured using ^18^F-fluoro-deoxyglucose positron emission tomography (^18^F-FDG PET) (15). Radiotracer concentrations in specific regions of the brain reflect disease-related patterns of neurofunctional changes, meaning that these clinical changes can therefore inform disease diagnosis. ^18^F-FDG PET is used to measure an index of cerebral glucose metabolism for several neurodegenerative brain diseases (16, 17), such as Alzheimer's disease (18), spastic CP (19), and Parkinson's disease (20, 21). PET has recently been introduced for monitoring gene therapy for CP (19, 22) and supranuclear palsy (23), suggesting that it is helpful in the differential diagnosis and therapy improvement of CP (24, 25). ^18^F-FDG PET/CT results demonstrate differential cell metabolism-related activation and deactivation patterns in the motor areas of children with spastic diplegic CP (26). To date, regional cerebral FDG uptake has been analyzed using several different techniques, and there is no standard approach to the early diagnosis of CP without structural abnormalities. None of the studies described above have explored the correlation of motor function with cerebral glucose metabolism, especially in the cortex, the impairment of which is a fundamental feature of CP.

Therefore, we investigated the distinctive patterns of cerebral metabolism changes in children with retrospectively diagnoses of CP, but who exhibited no structural abnormalities on MRI. This trial study establishes a reference dataset that can be compared with single patient datasets. We hypothesized cerebral glucose metabolism in the brain would correlate with GMFCS scores in CP patients. If so, these findings might eventually be helpful in the clinical diagnosis of individual CP patients.

## Materials and methods

### Human subjects

Medical records of thirty-one CP patients (2–4 years of age; 23 male, 8 female) were recruited from the Taihe Hospital. The inclusion criteria were diagnosed cases of CP caused by transient injury, without clinical complications of epilepsy. During clinical evaluation, the motor, linguistic, and cognitive functions of patients were assessed using the GMFCS (12) as our previous study (27), with all patients in the study classified as level II–V (Table 1). All subjects undergoing conventional MRI showed no structural brain abnormalities with regard to their clinical findings. The exclusion criteria: patients with acute infections, major organs (e.g., liver or kidney) dysfunction, and other space occupying lesion in brain. The present study was approved by the Research Ethics Committee at Taihe Hospital (No. 20140801).

**Table 1:** Clinical features of the studied cohort (total number = 31).

**Demographic characteristics**	**Demographic groups**	**Number of patients**
Sex	Male	23
	Female	8
Age	<3	10
	3–4	15
	>4	6
GMFCS	Level II	5
	Level III	6
	Level IV	11
	Level V	9

GMFCS, the Gross Motor Function Classification System.

### Protocol for ^18^F-FDG PET acquisition

Images of cerebral glucose metabolism were acquired using an ^18^F-FDG PET/CT system (Biograph mCT-64; Siemens Healthcare). Most patients fasted for at least 6 h, but some fasted for only 4 h for the risk of hypoglycemia. They had free access to water until the start of the imaging. Children were placed supine on the scan table after sedation in the absence of any factors that could affect their cerebral metabolism before the scan. FDG was administered intravenously at a dose of 3.5 MBq/kg. The head position of the patient was monitored 45 min later, with the monitoring lasting 10 min. The PET images were reconstructed using a posterior-based three-dimensional iterative algorithm. After which, followed by iteratively attenuation correction with a low-dose CT image (28). Then standardized uptake values (SUVs) were normalized to the amount of injected ^18^F-FDG, patient body weight.

### Evaluation of PET data

Image processing was performed, and the results were visually evaluated by two experienced nuclear medicine physicians blinded to the final clinical diagnosis. Quantitative analysis of axial, coronal, and sagittal slices of the brain PET images was performed using Scenium software (Siemens Healthcare), as previously described (29). This software uses a standard template containing multiple 3D anatomical regions of interest (ROIs) to find an accurate match for each cortical structure. To analyze the concentration of tracer in the brain, we quantified the activity concentration for the following nine regional volume of interests (VOIs): basal ganglia, central region, cerebellum, cingulate and paracingulate gyri, frontal lobe, mesial temporal lobe, occipital lobe, parietal lobe, and temporal lobe. The ^18^F-FDG concentration in the whole brain was also calculated as a reference. Averages of the mean standardized uptake value (SUVmean) and the asymmetry index [2 × 100 (*L*–*R*)/(*L* + *R*)] were calculated in each of the selected brain regions. SUVmean values of all ROIs were divided by the mediastinal blood pool in order to correct for inter-individual variability.

### Statistical analysis

SPSS software (version 24.0; SPSS for Windows) was used to perform the statistical analysis. Spearman's rho test was used to evaluate correlations between the SUVmean and GMFCS levels in patients with CP. The regional SUVmean values were compared between the left and right cerebral hemispheres. Comparisons between two groups were determined using two sample *t*-test for normally distributed data, otherwise using a non-parametric Mann Whitney *U*-test. Significance levels were set at *p* < 0.05.

## Results

### Characteristics of patients

The demographics of the study population and the GMFCS level results are presented in Table 1. The patients with CP comprised 23 males and 8 females. The median age of them was 3 years, with a range of 2–4 years at the time of ^18^F-FDG PET imaging. All patients were assessed clinically using the GMFCS, with the levels assigned ranging from II–V and the majority of patients showing severe CP (GMFCS level IV–V, 64.5%).

### Asymmetries in brain glucose metabolism

**Figure 1:**
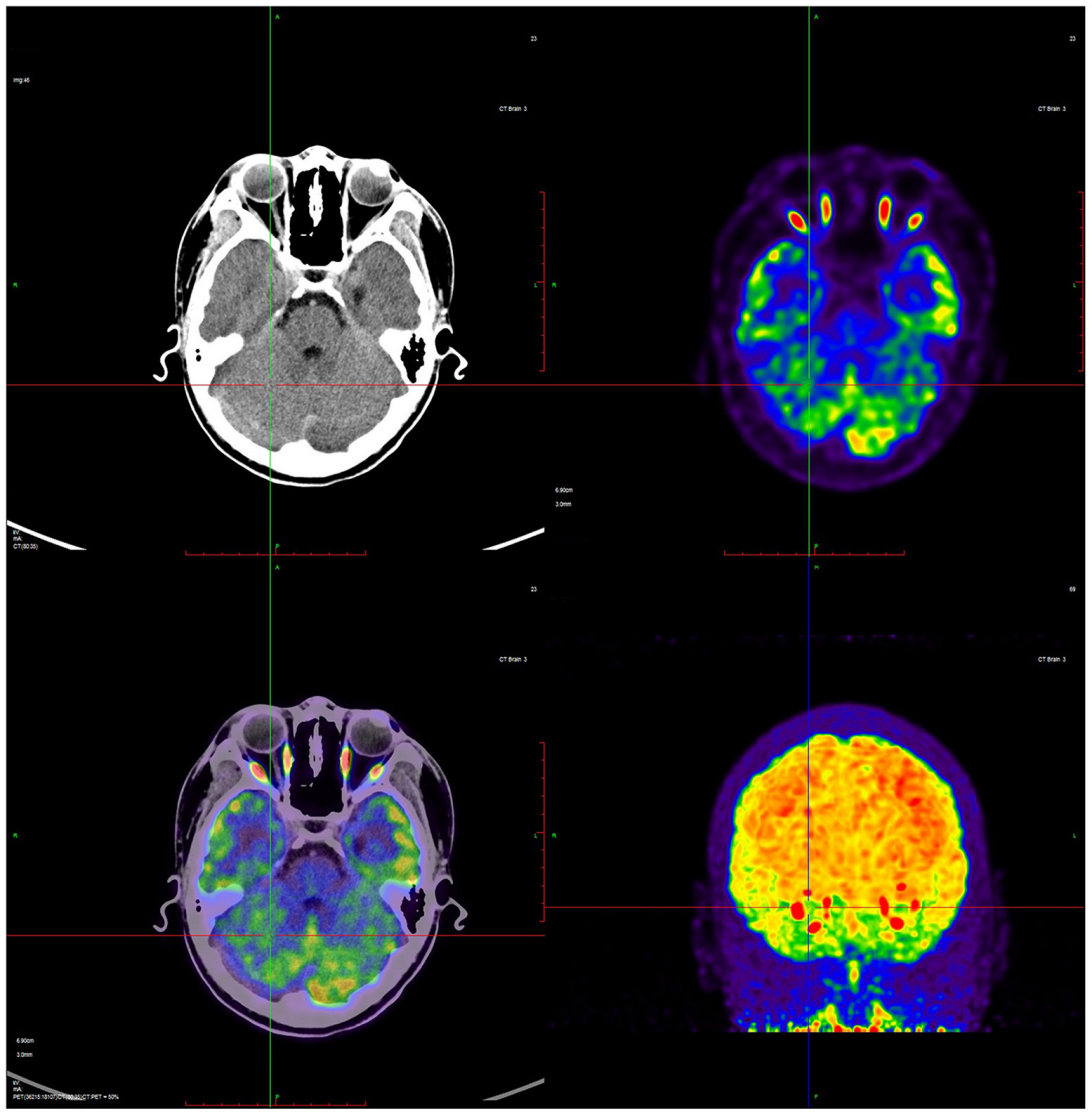
A representative ^18^F-FDG brain PET image from a CP child showed the relative higher glucose metabolism in left cortex. Red color indicates relatively high glucose metabolism and blue relatively low in comparison to corresponding regions.

Based on ^18^F-FDG PET imaging, regional neuronal activity was determined in each of the patients with CP. The levels of metabolic activity for each ROI are presented in Table 2. In the analysis of regional metabolic activity, a left > right asymmetry was more frequently observed. Figure 1 shows a typical brain ^18^F-FDG PET image from a CP patient demonstrating left > right asymmetry. In the individual analysis, 24 (77.4%), 26 (83.8%), 22 (71.0%), and 23 patients (74.2%) exhibited left > right asymmetry of glucose metabolism in the central region, cerebellum, frontal lobe and parietal lobe, respectively. Statistical analyses revealed that levels of glucose metabolism in the left central region, cerebellum, frontal lobe, and parietal lobe were significantly higher than those of the corresponding regions in the right hemisphere (*p* < 0.01).

**Table 2:** Cerebral metabolic ratio and asymmetry index in CP patients.

**Regions**		**Median**	**Interquartile range**	**Asymmetry index (** ***n*** **)**		**Asymmetry index (%)**	Left vs. right*
				**L>R**	**L ≤ R**		
Basal ganglia	L	4.54	3.49–5.03	13	18	−0.33 (−6.92 to 2.6)	0.40
	R	4.32	3.74–5.04				
Central region	L	4.93	4.38–5.69	24	7	3.58 (0.2–5.93)	<0.001*
	R	4.71	4.25–5.34				
Cerebellum	L	3.10	2.52–3.8	26	5	5.48 (1.34–8.96)	0.002*
	R	2.98	2.53–3.67				
CPG	L	4.88	4.34–5.26	19	12	1.4 (−1.32 to 4.12)	0.13
	R	4.52	4.26–5.39				
Frontal lobe	L	4.85	4.25–5.58	22	9	2.28 (−1.03 to 3.98)	0.003*
	R	4.74	4.23–5.63				
Mesial temporal lobe	L	3.21	2.75–3.52	14	17	−0.68 (−3.21 to 2.22)	0.60
	R	3.07	2.83–3.55				
Occipital lobe	L	4.99	4.43–5.51	10	21	−0.17 (−4.85 to 0.58)	0.13
	R	5.06	4.65–5.62				
Parietal lobe	L	5.28	4.44–5.69	23	9	1.78 (−0.24 to 3.92)	0.01*
	R	5.10	4.33–5.54				
Temporal lobe	L	4.67	4.13–5.21	17	14	0.56 (−1.54 to 2.69)	0.76
	R	4.83	4.02–5.51				

The data are presented as the medians marked with interquartile range. CPG, Cingulate and paracingulate gyri; L, left; R, right; Significance level: ^*^p < 0.05.

### Metabolic correlates of motor function

**Figure 2:**
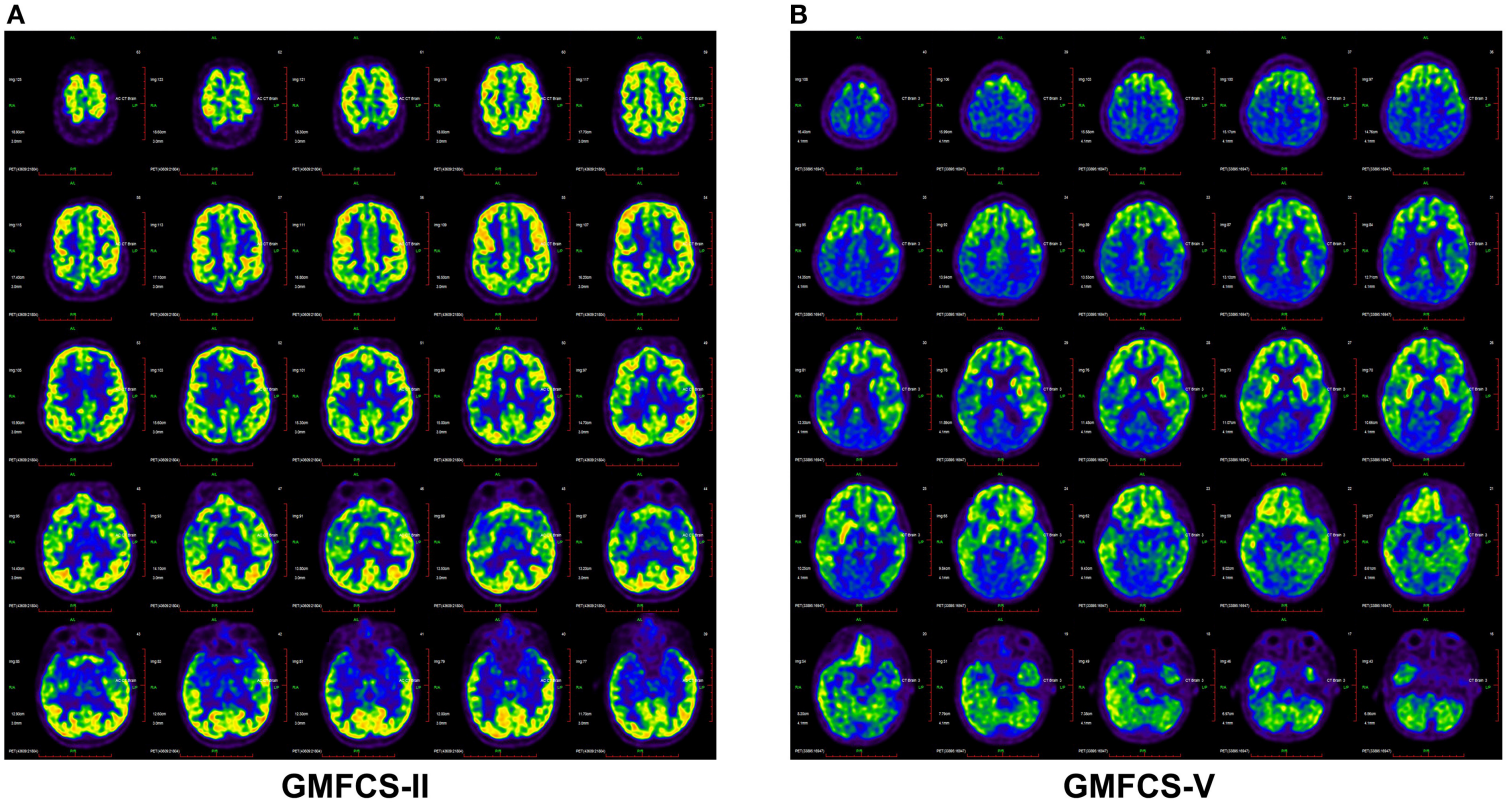
Brain ^18^F-FDG PET images demonstrated different metabolic activity of CP patients with GMFC level II **(A)** and V **(B)**. The CP patients with GMFC level V exhibited decreased activity compared with GMFC level II.

**Figure 3:**
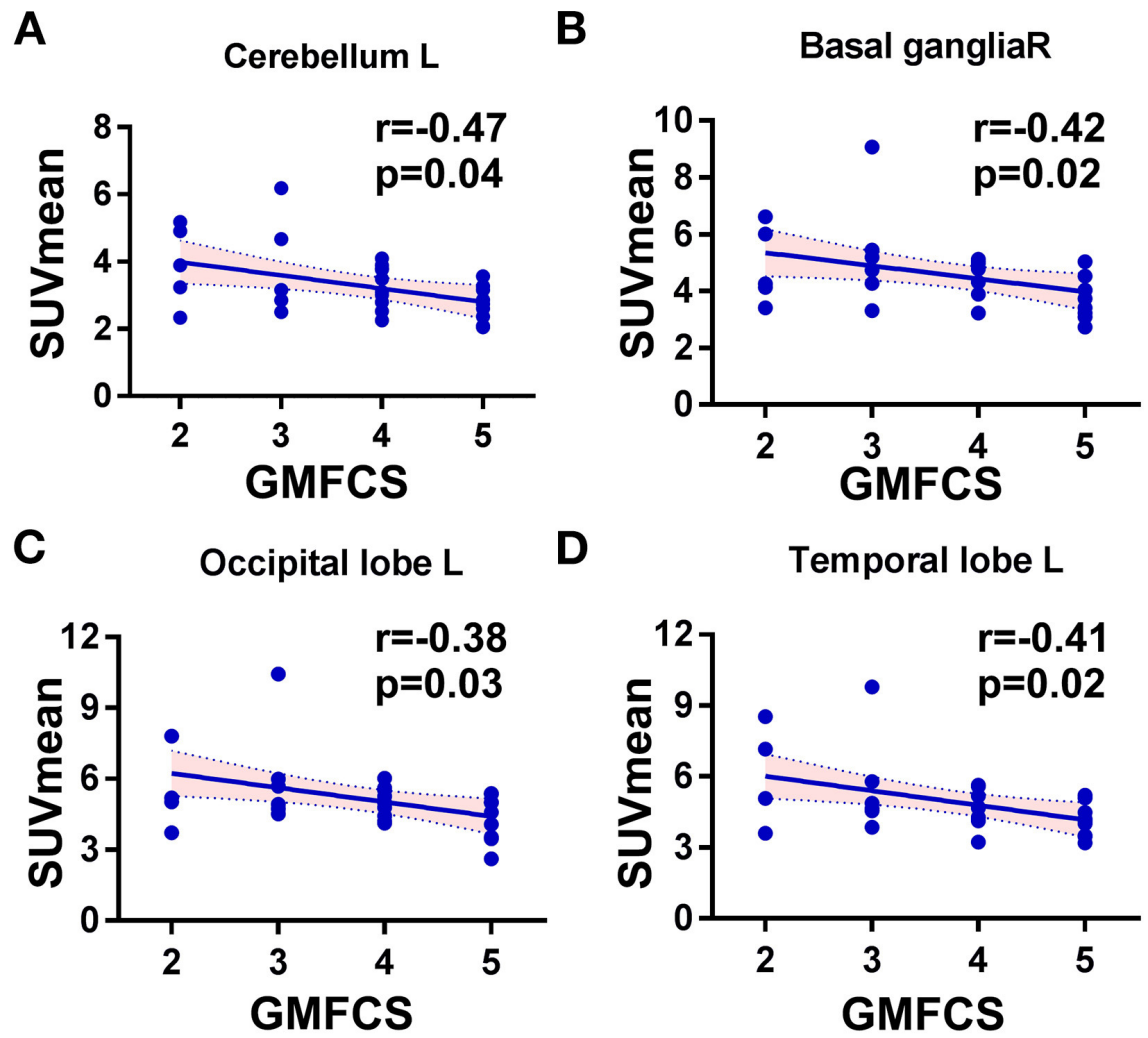
Scatter plots showing the significant correlations between GMFC levels and regional SUVmean in CP patients. **(A)** The cerebellum, left side: Spearman *r* = −0.47, *p* = 0.01. **(B)** The basal ganglia, right: Spearman *r* = −0.42, *p* = 0.02. **(C)** The occipital lobe, left side: Spearman *r* = −0.39, *p* = 0.02. **(D)** The temporal lobe, left side: Spearman *r* = −0.41, *p* = 0.03.

To investigate the potential association between regional metabolic activity and motor functioning, the children were divided into four groups according to their GMFCS levels. Figure 2 shows regional increases in the metabolic activity of patients with GMFCS level II compared to those with GMFCS level V. Based on the PET data, correlation analyses between regional SUVmean values and GMFCS scores were calculated and are summarized in Table 3 for each patient. As shown in Table 3, levels of motor function measured by the GMFCS were significantly negatively correlated with SUVmean in: bilateral basal ganglia (e.g., right: Spearman's *r* = −0.419, *p* = 0.02; mean: Spearman's *r* = −0.384, *p* = 0.03); left cerebellum (Spearman's *r* = −0.369, *p* = 0.01); left occipital lobe (Spearman's *r* = −0.388, *p* = 0.02); and bilateral temporal lobe (e.g., left: Spearman's *r* = −0.413, *p* = 0.03; mean: Spearman's *r* = −0.381, *p* = 0.02). Figure 3 shows scatter plots corresponding to these significant correlations from the entire study dataset. There were no other significant correlations in any other ROI.

**Table 3:** Association between the regional ^18^F-FDG uptake as obtained by volume of interest (VOI) analysis and the GMFCS levels of the CP patients (*n* = 31).

**Regions**		**II**	**III**	**IV**	**V**	Rho*
Basal ganglia	L	4.4 (3.5–5.3)	4.9 (4.1–6.2)	4.6 (4.4–5.1)	4 (3.4–4.3)	−0.29
	R	4.3 (3.8–6.3)	5 (4–6.4)	4.8 (4.3–5)	3.7 (3.2–4.3)	−0.419*
	Avg	4.3 (3.7–5.8)	5 (4–6.2)	4.8 (4.4–5)	3.6 (3.2–4.4)	−0.384*
Central region	L	5.8 (4.3–7.7)	5 (4.4–6.7)	4.9 (4.4–5.7)	4.9 (3.2–5.5)	−0.30
	R	6 (4.1–7.7)	4.8 (4.3–6.4)	4.7 (4.5–5.3)	4.4 (3.2–5.1)	−0.33
	Avg	5.9 (4.2–7.7)	4.9 (4.3–6.5)	4.9 (4.5–5.5)	4.6 (3.2–5.3)	−0.30
Cerebellum	L	3.9 (2.8–5)	3 (2.5–5.1)	3.1 (2.8–3.8)	2.7 (2.2–3.2)	−0.369*
	R	3.5 (2.6–4.4)	2.8 (2.4–4.6)	3.1 (2.6–3.7)	2.7 (2.4–3.2)	−0.22
	Avg	3.7 (2.7–4.7)	2.9 (2.4–4.8)	3.1 (2.7–3.8)	2.8 (2.4–3.2)	−0.28
Cingulate and paracingulate gyri	L	5.1 (4.3–7.2)	4.9 (4.3–6.5)	4.7 (4.6–5.3)	4.4 (3.2–5.2)	−0.32
	R	5.1 (4.1–7.1)	4.9 (4.1–6.7)	4.5 (4.3–5.3)	4.4 (3.3–5.2)	−0.31
	Avg	5.1 (4.2–7.2)	4.9 (4.2–6.6)	4.6 (4.4–5.3)	4.6 (3.3–5.2)	−0.32
Frontal lobe	L	5.1 (4.1–7.9)	5.1 (4.5–7.2)	4.9 (4.6–5.5)	4.4 (3.6–5.2)	−0.31
	R	5 (4.2–7.6)	4.9 (4.2–7)	4.7 (4.4–5.3)	4.2 (3.4–5.5)	−0.34
	Avg	5.1 (4.2–7.8)	5 (4.4–7.1)	4.8 (4.5–5.4)	4.3 (3.5–5.4)	−0.35
Mesial temporal lobe	L	4 (2.8–4.6)	3.2 (2.7–4.4)	3.2 (2.8–3.5)	2.8 (2.3–3.3)	−0.34
	R	4 (2.8–4.8)	3.2 (2.7–4.4)	3.2 (3–3.3)	2.9 (2.4–3.7)	−0.26
	Avg	4 (2.8–4.7)	3.2 (2.7–4.4)	3.2 (2.9–3.3)	2.8 (2.4–3.7)	−0.31
Occipital lobe	L	5.2 (4.4–7.8)	5.3 (4.7–7.1)	5 (4.4–5.5)	4.6 (3.5–5.2)	−0.388*
	R	5.2 (4.4–7.8)	5.3 (4.8–7.1)	4.9 (4.7–5.6)	4.9 (3.3–5.5)	−0.32
	Avg	5.2 (4.4–7.8)	5.3 (4.7–7.1)	4.9 (4.5–5.5)	4.5 (3.4–5.3)	−0.357*
Parietal lobe	L	5.4 (4.5–8.1)	5.3 (4.6–7.1)	4.9 (4.7–5.5)	4.9 (3.6–5.6)	−0.26
	R	5.3 (4.4–7.8)	5.1 (4.5–6.7)	4.8 (4.5–5.5)	4.4 (3.5–5.6)	−0.25
	Avg	5.3 (4.4–7.9)	5.2 (4.6–6.9)	4.9 (4.4–5.6)	4.7 (3.5–5.6)	−0.25
Temporal lobe	L	5.1 (4.3–7.9)	4.8 (4.4–6.8)	4.7 (4.2–5.6)	4.1 (3.5–4.8)	−0.413*
	R	5.4 (4.2–7.7)	4.7 (4.3–6.8)	4.6 (4.3–5.5)	4 (3.4–5.3)	−0.34
	Avg	5.3 (4.3–7.8)	4.7 (4.4–6.8)	4.7 (4.3–5.6)	4.1 (3.4–5)	−0.381*

The data are presented as the medians marked with interquartile range. The correlation data shown here are Spearman correlation coefficients r. VOI, volume of interest; CPG, Cingulate and paracingulate gyri; L, left; R, right; Avg, Average; Significance level: ^*^p < 0.005.

**Figure 4:**
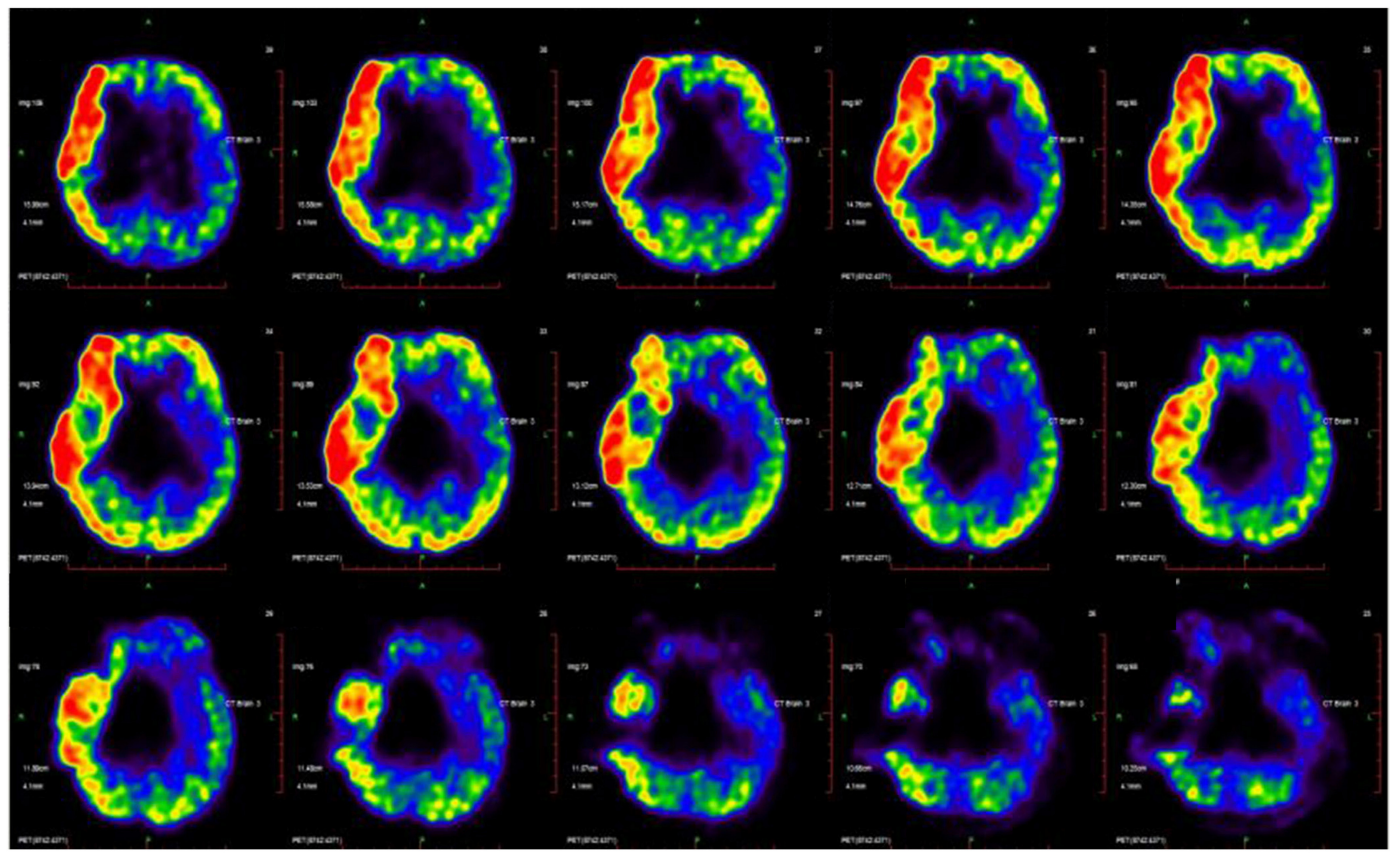
CP's disease-related pattern identified by analysis of FDG PET scans from 31 CP patients. The glucose metabolism in the left cerebellum relative higher than the right hemisphere.

The dataset as whole revealed that the left cerebellum showed higher metabolic activity than the right in most patients (83.8%) (Figure 4), with levels of metabolic activity also negatively correlated with GMFCS scores (Spearman's *r* = −0.47, *p* = 0.01). Neural activity in this brain region is known to correlate with different aspects of motor function.

## Discussion

It is important to diagnose patients with CP as soon as possible to enable early interventions and accurate evaluation of any therapeutic effects. We investigated the typical cerebral glucose metabolism of patients with CP using brain ^18^F-FDG PET imaging, although the patients showed no apparent evidence of structural abnormalities on brain MRI. We found metabolic asymmetries, with a left > right asymmetry in several brain regions in the central region, cerebellum, frontal lobe, and parietal lobe. This marked asymmetric pattern largely reflects physiological developmental changes, rather than structural abnormalities or the clinical presentation of CP. Despite the small size of our patient sample, we were able to define CP-specific brain metabolic patterns based on ^18^F-FDG PET imaging. Our results indicate a significant correlation between regional glucose metabolism and the extent of motor impairment, as assessed by GMFCS level. These findings are encouraging enough for our approach to be developed further to support the clinical diagnosis of cases of CP without structural abnormalities.

Neuroimaging studies contribute to identifying brain abnormalities and improving the accuracy of CP diagnosis. Previous studies have reported structure–function associations between clinical CP and patterns in MRI data, with structural abnormalities in children with CP corresponding to white matter injury (30) or gray matter injury (31). However, ~15% of children show no abnormalities in their MRI scans. According to Mohan et al. (32), brain single photon emission-computed tomography (SPECT) has also been applied to the diagnosis of CP and can detect abnormalities in the perfusion of cortical regions, subcortical nuclei, and cerebellar regions. Compared with SPECT, MRI, and other anatomical imaging techniques, PET scanning is more sensitive at detecting functional abnormalities associated with CP (22). The molecular imaging modality and brain metabolism measured using ^18^F-FDG PET scanning reflects regional neuronal activity. Asymmetries in glucose metabolism have also typically been observed in epilepsy (33) and corticobasal degeneration (34), and may help clinicians to diagnose these brain diseases. The present study was the first to use PET to identify typical brain metabolic patterns in CP patients without MRI abnormalities.

A prior PET study revealed thalamic and parieto-occipital-temporal cortical hypometabolism in spastic diplegic patients without apparent structural abnormality (35). In accordance with the PET and SPECT findings of this earlier study, we also observed that children with CP exhibited relatively higher levels of glucose metabolism in several cortical regions. Our study showed asymmetrical metabolism in a number of cortical regions, with left > right asymmetries most prominent in the parietal lobe, hemisphere, central region, cerebellum, and frontal lobe. In contrast, levels of the metabolism in the occipital-temporal cortices were not significantly increased compared with those in the contralateral brain regions.

During the progression of CP, children with the disease but without structural brain abnormalities continue to experience motor and cognitive dysfunction, and language and neurobehavioral impairments (2, 7). In general, the extent of metabolic activity changes measured by ^18^F-FDG PET imaging correlates with the clinical status and severity of neurodegenerative disease (36, 37). However, few studies to date have investigated brain metabolism in CP correlated with motor function. Fowler et al. found a negative correlation of metabolic activity with selective voluntary motor control in the cerebellum (19). We used the GMFCS in our study to quantify motor function, and observed a significant correlation between GMFCS scores and regional cortical hypometabolism in regions such as the basal ganglia, left cerebellum, left occipital lobe, and temporal lobe. Cerebral metabolic patterns might also be helpful for evaluating the differential effects of treatments on motor function. The efficacies of different treatments have been assessed according to the clinical function and cerebral glucose metabolism activity in children with CP based on the grading of gross motor function (27, 38).

One limitation of the study was the small patient sample size, which did not allow us to collect enough data to cover all of the GMFCS levels. However, similar PET studies have included even fewer participants with CP (19, 38). Another limitation is that we did not include healthy children as a control group in the study. Thus, although we investigated cerebral metabolic patterns in children with CP who had varying levels of motor function, the corresponding patterns in healthy children are unknown. Concerning the effects of radiation, further research is suggested to minimize ionizing radiation to children. This study would have been strengthened by examining the correlations between patients and healthy controls using ^18^F-FDG PET scanning.

In conclusion, individual levels of glucose metabolism in the cerebral cortex, cerebellum, and basal ganglia correlated well with the severity of clinical motor dysfunction. The diagnosis of CP is based on an interview, observation, and neurological examination. The typical cerebral metabolic patterns of CP identified here based on brain ^18^F-FDG PET imaging will help clinicians to differentiate between CP associated with different levels of motor impairment. Compared to MRI, it is expensive examination, which limits its popularization. But this is particularly true for cases of CP in which microstructural abnormalities could not detected by MRI. A major benefit of this study is that the findings can be used as a reference CP patient dataset, which might ultimately contribute to diagnosing CP in its early stages. The matabolic pattern could be validated and conducted on the animal model in the further study.

## Data availability statement

The raw data supporting the conclusions of this article will be made available by the authors, without undue reservation.

## Ethics statement

The studies involving human participants were reviewed and approved by the Research Ethics Committee at Taihe Hospital. Written informed consent to participate in this study was provided by the participants' legal guardian/next of kin.

## Author contributions

RW, JG, and ZP contributed to the study design. HW, HZ, and FT contributed to the data acquisition. YG and RW simulated, analyzed the data, and drafted the manuscript. DZ, YY, and YC were involved in data interpretation. ZP and YG contributed to the critical revision of the manuscript. All authors revised the manuscript, read, and approved the final manuscript.

## Funding

This work was supported by the Hubei Province's Outstanding Medical Academic Leader program, the Foundation for Innovative Research Team of Hubei Provincial Department of Education (No. T2020025), the General Project of Hubei Provincial Department of Education (No. B2021160), the Hubei Provincial Department of Science and Technology Innovation Group Program (No. 2019CFA034), Innovative Research Program for Graduates of Hubei University of Medicine (No. YC2020011), and the Key Discipline Project of Hubei University of Medicine.

## Conflict of interest

The authors declare that the research was conducted in the absence of any commercial or financial relationships that could be construed as a potential conflict of interest.

## Publisher's note

All claims expressed in this article are solely those of the authors and do not necessarily represent those of their affiliated organizations, or those of the publisher, the editors and the reviewers. Any product that may be evaluated in this article, or claim that may be made by its manufacturer, is not guaranteed or endorsed by the publisher.
